# Structural Evolution Leading to the Thermosalient Phase Transition of Oxitropium Bromide

**DOI:** 10.3390/molecules30051107

**Published:** 2025-02-28

**Authors:** Teodoro Klaser, Jasminka Popović, Ivor Lončarić, Željko Skoko

**Affiliations:** 1 Department of Physics, Faculty of Science, University of Zagreb, Bijenička 32, 10000 Zagreb, Croatia; tklaser@phy.hr; 2 Department of Physics, University of Trento, Via Sommarive 14, 38123 Trento, Italy; 3 Ruđer Bošković Institute, Bijenička 54, 10000 Zagreb, Croatia; jpopovic@irb.hr (J.P.); iloncar@irb.hr (I.L.)

**Keywords:** thermosalient effect, phase transition, negative thermal expansion, thermal anisotropy, PXRD, DFT, oxitropium bromide

## Abstract

This study investigates the thermosalient effect in oxitropium bromide, with a focus on the role of anisotropic thermal expansion, elastic properties, and sound propagation in driving this phenomenon. Variable-temperature X-ray powder diffraction (VTXRPD) revealed significant anisotropic thermal expansion, including negative thermal expansion (NTE) along the c-axis in the low-temperature Form A. Density functional theory (DFT) calculations were used to analyze elastic properties of oxitropium bromide and confirmed that it does not exhibit negative compressibility, emphasizing thermal anisotropy as the primary factor in the phase transition. Studies of elastic constants and sound propagation demonstrated a preferred pathway for energy transfer along the *z*-direction, enabling rapid strain release during the phase transition. These findings confirmed that the thermosalient effect arises from cooperative molecular motion, resulting in an abrupt and energetic transformation driven by the interplay of structural anisotropy and elastic properties.

## 1. Introduction

Thermosalient materials, colloquially dubbed “jumping crystals”, are a remarkable class of materials that exhibit macroscopic mechanical motion as a response to thermal stimuli. This unique and quite rare behavior, characterized by abrupt movements such as jumping, flipping, rolling, or breaking, arises from a thermally induced phase transition, quite often a single crystal to single crystal. The thermosalient effect was first discovered serendipitously in 1983 through the pioneering work of Etter and Siedle, who observed a remarkable behavior in (phenylazophenyl)palladium hexafluoroacetylacetonate [1]. These materials have garnered significant attention in recent years, not only for their scientific intrigue but also for their potential applications in smart materials, actuators, sensors, and energy conversion systems [2,3,4,5,6,7,8,9,10,11,12,13,14,15,16,17,18,19,20,21,22,23,24,25,26,27,28,29,30,31,32,33,34,35,36,37,38,39,40,41,42,43,44,45,46,47,48,49,50,51,52,53,54,55].

Thermosalient behavior is rooted in the intrinsic properties of the crystal lattice. When exposed to temperature changes (heating or cooling), the material undergoes a structural transformation, often involving a shift between polymorphic forms. These phase transitions are typically first order in nature [16,40], which means that they are associated with discontinuities in properties such as density, volume, or enthalpy. During heating or cooling, mechanical stress accumulates within the crystal lattice due to anisotropic expansion or contraction. When this stress surpasses a critical threshold, it is released explosively, resulting in dramatic macroscopic motion [12,14,16,20,50,56]. As stated, the expansion/contraction of the unit cell is anisotropic, and most of the thermosalient materials exhibit uniaxial or even biaxial negative thermal expansion (NTE). This phenomenon is rare and highly specific to certain molecular crystals. A defining feature of thermosalient materials is their ability to undergo these transitions reversibly, allowing the crystal to repeatedly “jump” or deform in response to heating and cooling cycles. The reversibility and robustness of these transformations are key to their potential for practical applications [16,17,20,23,41].

This alluring effect is driven by molecular and crystallographic factors. At the molecular level, the materials often consist of small, rigid molecules that pack in a highly ordered yet metastable manner. The transition between phases typically involves subtle rearrangements of these molecules, such as changes in hydrogen bonding, π–π stacking, or van der Waals interactions. These rearrangements alter the lattice dimensions, creating internal stress. Crystallographically, the thermosalient effect is facilitated by specific symmetries and anisotropies within the crystal [8,16]. Typically, thermosalient materials possess layered or needle-like structures since they allow for directional stress propagation [16].

Thermosalient materials hold promise in various technological domains due to their ability to convert thermal energy into mechanical motion. One potential application lies in the development of thermal actuators, which can operate without the need for external power sources. Such devices could be used in robotics, microfluidics, or adaptive systems that respond to environmental changes. Another exciting avenue is energy harvesting. By coupling thermosalient materials with piezoelectric components, it may be possible to harness the mechanical energy released during phase transitions and convert it into electrical energy. This approach could contribute to the development of sustainable energy solutions, particularly in low-power devices. In addition, thermosalient crystals are being explored as sensors for temperature changes. Their visible and dynamic responses to thermal stimuli make them ideal candidates for applications requiring precise thermal monitoring.

Despite their potential, significant challenges persist in the study and application of thermosalient crystals. A major limitation is the scarcity of materials exhibiting this behavior, as only a few known compounds display thermosalient effects. To address these challenges, researchers are focusing on understanding the molecular design principles that govern thermosalient behavior. Understanding and designing thermosalient materials require an interdisciplinary approach, combining insights from chemistry, physics, and engineering.

**Figure 1:**
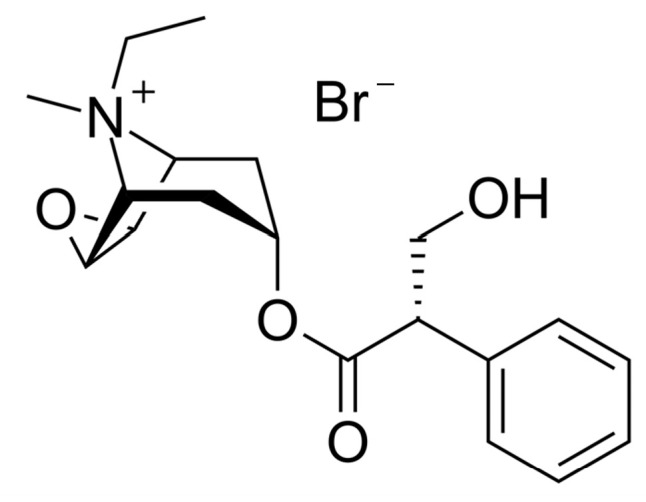
Chemical structure of OXTB.

The first systematic investigation of the thermosalient effect was conducted by Skoko et al. [8], who explored this phenomenon in the anticholinergic agent oxitropium bromide (OXTB) (Figure 1) using microscopy, as well as various thermal, spectroscopic, and structural techniques. This study revealed that the crystal jumping observed is a macroscopic manifestation of a highly anisotropic change in the cell volume and is occurring during the phase transformation of the low-temperature Form A to the high-temperature Form B at approximately 45 °C. This cell distortion is accompanied by a conformational change in the oxitropium cation, which results in an increased separation between the ion pairs in the lattice despite the cation and anion within each ion pair maintaining nearly identical distances. On a molecular level, the cation behaves like a molecular shuttle, consisting of two rigid components (an epoxy-aza-tricyclic-nonyl segment and a phenyl ring) connected by a flexible ester linkage. The structure of the rigid aza-tricyclic part remains largely unaffected by temperature, suggesting a mechanism in which the thermally accumulated strain is transferred via the ester bridge to the phenyl ring, triggering its rotation and thereby initiating the phase transition. It was suggested that the thermosalient phenomenon contains many elements of martensitic phase transformations in metals, which are characterized by cooperative, homogeneous movements, resulting in a change in the crystal structure.

Although significant research has been conducted on the thermosalient effect in OXTB, much of the focus has centered on the phase transition itself, with limited attention to the processes leading up to this transition [8]. This study builds upon previous work by investigating the thermosalient effect in OXTB, emphasizing the microstructural changes during heating that precede the phase transition, with special attention being devoted to the anisotropic nature of thermal expansion.

In a previous study [23], it was suggested that the interplay between thermal expansion and compressibility plays a critical role. This was demonstrated in another thermosalient compound, *N*’-2-propylidene-4-hydroxybenzohydrazide, where NTE was the consequence of the negative compressibility. Building on these insights, density functional theory (DFT) calculations were employed to examine the elastic properties of oxitropium bromide, including its bulk modulus and compressibilities, to determine whether this material also exhibits negative compressibility.

Additionally, the propagation of sound through the material was investigated in order to identify preferred directions for the transmission of elastic waves and energy, which are necessary for the occurrence of the thermosalient phase transition.

## 2. Results

### 2.1. In Situ Variable-Temperature X-Ray Powder Diffraction

**Figure 2:**
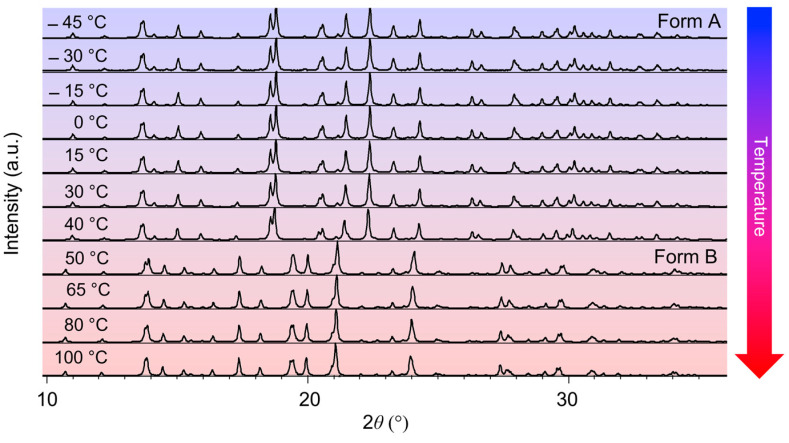
In situ VTXRPD Form A and Form B in the temperature range of T = −45 to 100 °C.

Temperature-induced structural changes of OXTB low-temperature Form A and high-temperature Form B were investigated by the in situ X-ray powder diffraction in the temperature range of −45 to 140 °C. This interval was chosen to include wide enough temperature ranges before and after the thermosalient phase transition (around 45 °C [8]) to obtain reliable data about structural changes leading to and after the phase transition. The VTXRPD patterns at selected temperatures are shown in Figure 2.

**Figure 3:**
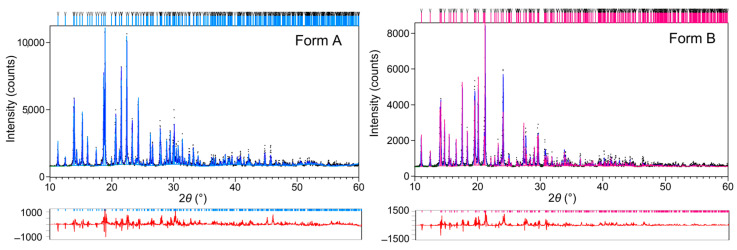
Rietveld structure refinements of OXTB at 40 °C and 50 °C. Experimental data are provided as black line, and calculated diffraction pattern is provided as blue line. The difference curve is provided below in red. Sky blue and magenta vertical lines represent positions of Bragg reflections of Form A and B, respectively.

It is obvious from Figure 2 that pronounced structural change caused by the phase transition of OXTB Form A and Form B occurs in the temperature range of 40–50 °C, as has already been shown in [8]. XRD patterns at 40 °C indicate that OXTB exists entirely in FORM A. However, at 50 °C, it transforms into FORM B. Rietveld structure refinements of OXTB Form A and Form B at T = 40 °C and T = 50 °C, respectively, are shown in Figure 3.

**Figure 4:**
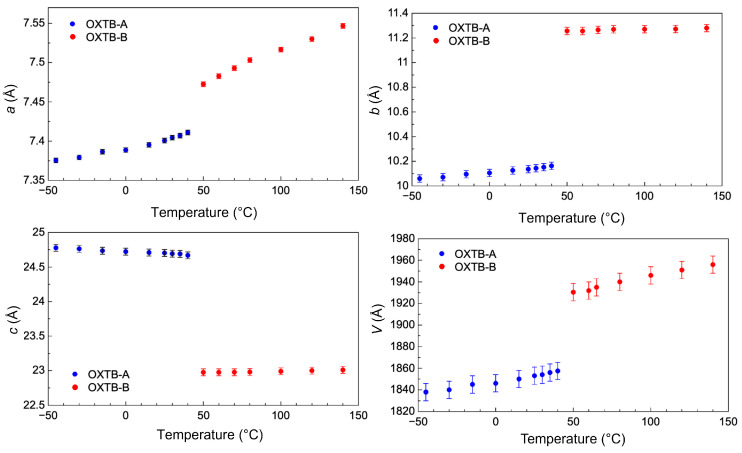
Unit-cell parameters of OXTB as a function of temperature determined by in situ VTXRPD. Blue and red rectangles denote Forms A and B, respectively.

Figure 4 shows the changes in the unit-cell parameters of OXTB Form A and Form B as a function of temperature determined by in situ VTXRPD. Blue and red rectangles denote Forms A and B, respectively.

Based on the refined lattice parameters, the thermal expansion coefficients *α* for OXTB Form A and Form B along principal axis *i* = 1, 2, and 3 were calculated and shown in Table 1 and Table 2.

**Figure 5:**
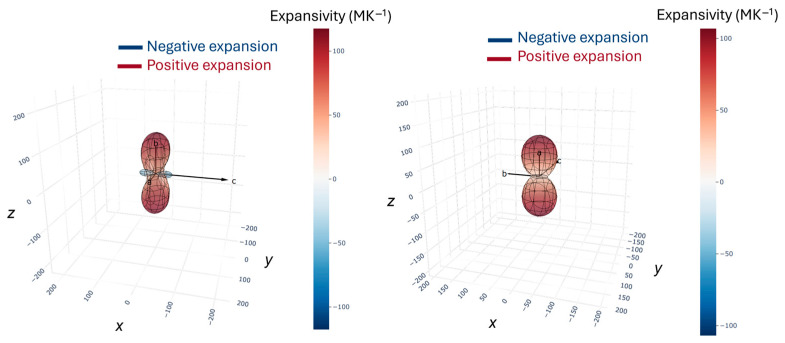
Thermal expansivity indicatrix of OXTB Forms A and B determined from in situ variable-temperature single crystal and powder X-ray diffraction.

Corresponding thermal expansivity indicatrix for OXTB Form A and Form B along the principal axis *x_i_* (*i* = 1, 2 and 3) are visualized in Figure 5.

It is essential to emphasize the notably high positive thermal expansion coefficients *α* along the crystallographic directions *a* and *b* for OXTB Form A. Typically, the thermal expansion coefficients for molecular crystals range from $0\;to\;20\times 10^{-6}\;K^{-1}$, but the values for OXTB Form A are up to five times higher than this usual maximum. For example, along the *b*-axis, the coefficient reaches $\alpha_{b}=117\times 10^{-6}\;K^{-1}$. The thermal expansion along the *a*-axis is also significant, with $\alpha_{a}=55\times 10^{-6}\;K^{-1}$ more than twice the typical maximum value.

A particularly notable discovery, made through in situ diffraction experiments, is the negative thermal expansion along the *c*-axis in OXTB Form A, where the corresponding coefficient is $\alpha_{c}=-47\times 10^{-6}\;K^{-1}$.

In contrast, the high-temperature Form B exhibits positive thermal expansion along all axes, with the coefficients presented in Table 2. Interestingly, the thermal expansion coefficients in the high-temperature form are considerably lower than those in the low-temperature form. While still high, the values for the *b* and *c* axes ($\alpha_{b}=21\times 10^{-6}\;K^{-1}$, $\alpha_{c}=18\times 10^{-6}\;K^{-1}$) fall within the typical range for molecular crystals. However, the thermal expansion remains highly anisotropic due to the exceptionally high value along the *a*-axis ($\alpha_{a}=104\times 10^{-6}\;K^{-1}$), which is nearly double the value observed in the low-temperature form.

As previously mentioned, the anisotropy of the crystal lattice’s thermal expansion is a critical prerequisite for the thermosalient transition. Notably, many known thermosalient compounds exhibit uniaxial or biaxial NTE. NTE is a rare phenomenon where materials contract upon heating, in contrast to the typical expansion behavior observed in most materials. This contraction arises from specific structural or electronic mechanisms that dominate in certain materials. The rarity of NTE stems from the fact that most materials expand when heated due to the anharmonic nature of atomic vibrations in their crystal lattices. Despite its rarity, NTE continues to attract significant research interest due to its unique properties and wide range of potential applications.

The transformation strain of the phase transition was calculated using the measured lattice constants. The transformation strain along the *b*-axis and *c*-axis is extremely large and anisotropic, amounting to 11% along the *b*-axis (tensile) and 7% along the *c*-axis (compressive). The overall volume change during the thermosalient phase transition is 4%. Such large values of the transformation strain are possibly the main reason for such a sudden and violent phase transition.

### 2.2. Elastic Properties of Oxitropium Bromide

Diffraction experiments provided valuable data on the thermally induced structural behavior of OXTB. Additionally, theoretical DFT calculations were performed to investigate the elastic properties of the system, including bulk modulus and compressibilities. This approach enables the study of anisotropy in the system and facilitates the analysis of the relationship between thermal and elastic properties, allowing comparisons with other thermosalient materials. The elastic constants and elastic compliance constants for OXTB Form A, calculated using the method described in Section 2.2, are presented in Equations (1) and (2).

Elastic constants *C_ij_* for OXTB Form A:

(1) $$C_{ij}=\left[\begin{array}{cccccc} 300 & 125 & 156 & 0 & 0 & 0\\ 125 & 271 & 73 & 0 & 0 & 0\\ 156 & 73 & 438 & 0 & 0 & 0\\ 0 & 0 & 0 & 177 & 0 & 0\\ 0 & 0 & 0 & 0 & 127 & 0\\ 0 & 0 & 0 & 0 & 0 & 114 \end{array}\right]Kbar$$

Elastic compliance constants *S_ij_* for OXTB Form A:

(2) $$S_{ij}=\left[\begin{array}{cccccc} 4.83 & -1.84 & -1.41 & 0 & 0 & 0\\ -1.84 & 4.56 & -0.102 & 0 & 0 & 0\\ -1.41 & -0.102 & 2.80 & 0 & 0 & 0\\ 0 & 0 & 0 & 5.66 & 0 & 0\\ 0 & 0 & 0 & 0 & 7.86 & 0\\ 0 & 0 & 0 & 0 & 0 & 8.77 \end{array}\right]{Mbar}^{-1}$$

Compressibility along the principal axes *x_i_* (*i* = 1, 2, and 3) was calculated from the elastic compliance constants and are presented in Table 3.

**Figure 6:**
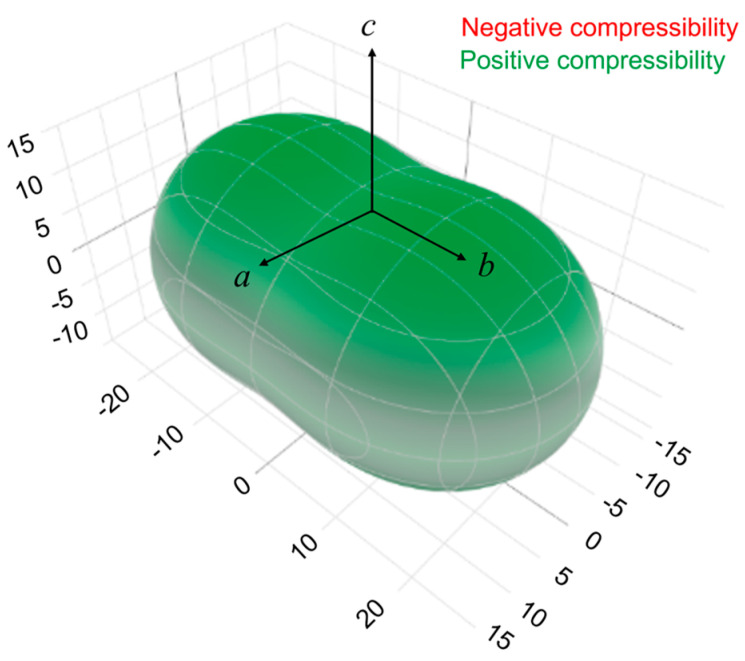
Linear compressibility indicatrix for OXTB Form A determined from DFT stiffness matrix calculations.

The corresponding compressibility indicatrix for OXTB Form A along the principal axis *x_i_ (i* = 1, 2, and 3) is visualized in Figure 6.

DFT calculations revealed that OXTB Form A exhibits positive compressibility in all three directions, with relatively isotropic changes in the unit-cell dimensions, despite displaying highly anisotropic thermal expansion, which is negative along the *c*-axis. This contrasts with thermosalient *N*’-2-propylidene-4-hydroxybenzohydrazide, where NTE along the *c*-axis was directly coupled with negative compressibility in the same direction. For OXTB, the thermal expansion behavior does not appear to stem from its elastic properties. Notably, while NTE prior to the thermosalient phase transition is a shared characteristic of both *N*’-2-propylidene-4-hydroxybenzohydrazide and OXTB, the occurrence of the thermosalient effect does not require negative compressibility, underscoring that it is not a critical factor for the manifestation of the jumping phenomenon.

The obtained values for linear compressibility $\left(K_{a}=15.8\;{TPa}^{-1},K_{b}=26.2\;{TPa}^{-1},K_{c}=12.9\;{TPa}^{-1}\right)$ fall in the lower range of the typical range for the molecular crystals ($10–400\;{TPa}^{-1}$, [57,58]) and are comparable to those observed in metals (typically $3–25\;{TPa}^{-1}$ [59]).

### 2.3. Sound Propagation and Anisotropy in the Oxitropium Bromide

Thermosalient phase transitions, which are extremely brisk (almost instantaneous) and very energetic, necessitate a well-defined pathway for efficient energy transfer, ensuring the seamless propagation of the transformation throughout the material. That is why studies were further expanded to investigation of sound properties in OXTB Form A using the procedure reported in [60], where the elastic constants determined by DFT are used to predict the sound velocities in a material by solving the Christoffel equation.

**Figure 7:**
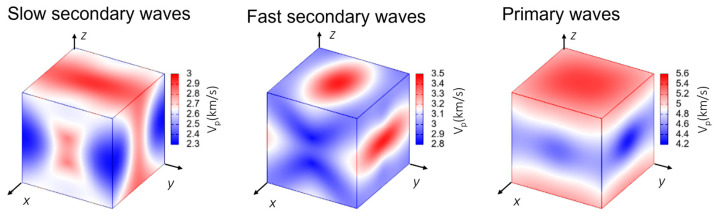
Phase velocity of waves through OXTB Form A.

To examine the propagation of waves in OXTB Form A, it is useful to visualize the behavior in a three-dimensional projection. The phase velocities of transverse modes (secondary waves) and longitudinal waves (primary waves, i.e., sound) for OXTB Form A are illustrated in Figure 7. Directions *x*, *y*, *z* correspond to the crystallographic axis *a*, *b*, *c*.

**Figure 8:**
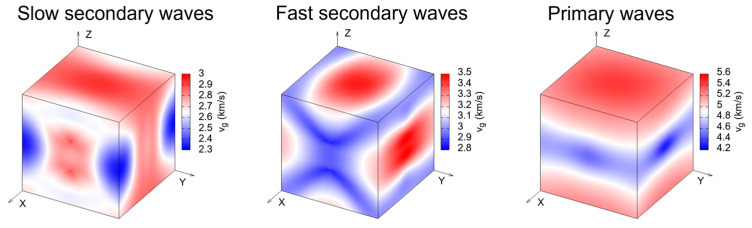
Group velocity of waves through OXTB Form A.

As shown in Figure 7, the phase velocity of the primary mode in OXTB is greater, whereas the secondary modes exhibit lower velocities. The absolute velocities indicate that all wave modes (primary, secondary, and slow secondary modes) propagate fastest along the *z*-direction compared to the *x-* and *y*-directions, with the primary modes being the fastest overall. The group velocity of sound waves is depicted in Figure 8.

**Figure 9:**
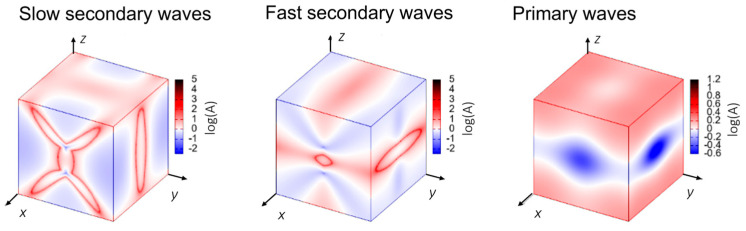
Enchantment factor of waves for oxitropium bromide.

Figure 8 demonstrates that the group velocity behaves similarly to the phase velocity, confirming that both primary and secondary waves propagate most rapidly in the *z*-direction. Figure 9 illustrates the enhancement factor of sound waves.

**Figure 10:**
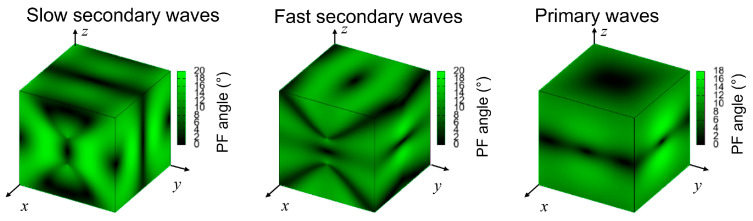
Waves power flow for OXTB Form A.

Figure 9 shows that the secondary modes, especially the slow secondary modes, exhibit a complex structure characterized by narrow bands of exceptionally high enhancement. In contrast, the primary mode demonstrates more moderate enhancement, primarily along the *z*-direction faces of the cube. Figure 10 presents the power flows for sound waves in OXTB.

Figure 10 illustrates that the power flow for primary waves is concentrated in the *z*-direction with minimal divergence, indicating a preferred path for the propagation of compressive waves and energy transfer. This suggests that the material is stiffer in this direction, allowing for the efficient release of accumulated strain energy. This is further supported by the DFT calculations, which show the smallest value of compressibility along the *c*-axis (Table 3). Notably, this direction aligns with the orientation in which OXTB exhibits NTE. Furthermore, the speed of sound waves in low-temperature Form A is approximately 5 km/s, which is comparable to that of metals and significantly higher than that of other molecular solids. This aligns with the compressibility calculations, where the obtained values fall within the range typical for metals and represent some of the lowest values observed for molecular crystals.

This analysis demonstrates that perturbations associated with the thermosalient effect propagate very quickly. The energy flow is focused rather than dissipated. Power flow is focused on primary (longitudinal) waves, which reflect the collective dynamics of the atomic lattice, where atoms move in sync with their neighbors, transmitting mechanical energy through the material. These findings fully support the hypothesis that the thermosalient effect arises from the cooperative movement of molecules within the crystal. This movement leads to an extremely energetic and rapid phase transition, with energy release occurring on a timescale of less than a millisecond, causing the crystals to leap off the surface.

The log–Euclidean anisotropy parameter *A^L^*, along with the indices *A^C^* and *A^U^*, as defined in [61], were calculated using a MATLAB 9.1 R2016b script designed to evaluate anisotropy in materials with well-known elastic constants. Briefly, *A^C^* is derived from the Voigt, Reuss, and Hill averages of the shear moduli and can be calculated for any crystal symmetry. However, due to the influence of shear and bulk moduli on the anisotropy of non-cubic crystals, *A^C^* is not entirely reliable. In contrast, the universal anisotropy index *A^U^* is applicable to all crystal symmetries. It is based on the fractional difference between the Voigt and Reuss bounds for both bulk and shear moduli.

However, like other anisotropy indices, *A^U^* provides a relative measure of anisotropy with respect to a limiting value. Due to this, the log–Euclidean anisotropy parameter *A^L^* was introduced, which provides an absolute measure of anisotropy for different materials. It is done by seeking the anisotropy distance between tensor average operation over all possible orientations of the crystallite’s elastic modulus and compliance. For further details on anisotropic indices, readers are encouraged to consult [61].

The calculated values for OXTB Form A are presented in Table 4.

The obtained values, when compared with those reported in the literature [57], are found to be comparable to those of metals such as thallium and platinum, which are known to exhibit some of the highest anisotropy levels.

## 3. Materials and Methods

Oxitropium bromide (C_19_H_26_BrNO_4_) used for the experiments was purchased from Sigma–Aldrich (Sigma–Aldrich, Steinheim, Germany) (>90%, HPLC). That sample was recrystallized from methanol/dichloromethane mixtures. For that purpose, the sample was first dissolved in a minimum quantity of methanol, to which a larger quantity of dichloromethane was subsequently added. After slow evaporation, the prismatic crystals of low-temperature Phase A were obtained.

### 3.1. Variable Temperature X-Ray Powder Diffraction

The crystal structure of the samples was thoroughly investigated by VTXRPD in the temperature range from −45 °C to 110 °C. The XRPD data were collected with a Bruker D8 Discover diffractometer (Bruker AXS GmbH, Karlsruhe, Germany) equipped with a LYNXEYE XE-T detector configured in a Bragg–Brentano geometry and Anton Paar high-temperature oven chamber HTK 1200N (Anton Paar GmbH, Graz, Austria). Data were collected in 2θ range of 10–50° with a step of 0.02° and Cu source with a wavelength of 1.540601 Å with Ni filter, 2.5° Soller slit, and fixed slit at 0.4 mm. The slit opening in front of the detector was 6.5 mm, and the detector opening was 1.3°, resulting in an integrating time per step of 25 s. The crystal structure was refined by the Rietveld method using X’Pert Highscore Plus software 3.0 (Malvern Panalytical, Almelo, The Netherlands). Refinement was conducted by using the split-type pseudo-Voigt profile function and the polynomial background model. Isotropic vibration modes were assumed for all atoms. During the refinement, a zero shift, scale factor, half-width parameters, asymmetry, and peak shape parameters were simultaneously refined. The structure of OXTB Form A, as reported by [4], was used as starting structural model for the Rietveld refinement for data collected at temperatures up to (and including) 40 °C, while the structure OXTB Form B, as reported by [8] al, was used for the Rietveld refinement of data collected for higher temperatures (50–110 °C). Thermal expansivity and linear compressibility indicatrices were obtained using PASCal v2.2.0, a web tool and open-source software package designed for analysis of non-ambient lattice parameter data [62].

Crystallographic data for OXTB Form A and Form B is as follows:

Form A: Raw Data Origin: BRUKER-binary V3 (.RAW); Scan Axis: Gonio; Start Position [°2Th.]: 10; End Position [°2Th.]: 60; Measurement Temperature [°C]: 40.00; Anode Material: Cu; K-Alpha1 [Å]: 1.54060; Diffractometer Type: Theta/2Theta D5000; Zero shift [°2Theta]: 0.001(2); R (weighted profile) [%]: 8.32327; Formula sum: C_76_._00_N_4_._00_O_16_._00_Br_4_._00_H_104_._00_; Formula mass [g/mol]: 1649.291; Density (calculated) [g/cm^3^]: 1.4775; Weight fraction [%]: 100.0; Space group (No.): P2_1_2_1_2_1_(19); a[Å]: 7.4109(8); b[Å]:10.1626(1); c[Å]: 24.6693(3).

Form B: Raw Data Origin: BRUKER-binary V3 (.RAW); Scan Axis: Gonio; Start Position [°2Th.]: 10; End Position [°2Th.]: 60; Measurement Temperature [°C]: 50.00; Anode Material: Cu; K-Alpha1 [Å]: 1.54060; Diffractometer Type: Theta/2Theta D5000; Zero shift[°2Theta]: 0.003(2); R (weighted profile) [%]: 5.91979; Formula sum: C_76_._00_N_4_._00_O_16_._00_Br_4_._00_H_104_._00_; Formula mass [g/mol]: 1649.291; Density (calculated) [g/cm^3^]: 1.4191; Weight fraction [%]: 100.0; Space group (No.): P2_1_2_1_2_1_(19); a[Å]: 7.4724(8); b[Å]:11.2567(1); c[Å]: 22.9746(3).

### 3.2. Density Functional Theory Calculations

For all DFT calculations, a plane-wave basis set code Quantum Espresso [63], with the GBRV pseudopotentials [64] and vdW-DF-cx [65,66] exchange-correlation functional, was used. The plane wave basis set cutoff for wave functions is 820 eV for stress calculations. In each calculation, atoms were relaxed until the change in the total energy was <10^−4^ eV, and all the forces were smaller than 2 × 10^−3^ eV/Å. Elastic constants were obtained using thermo pw extension [67] of Quantum Espresso package. First Brillouin zone was sampled by the Monkhorst-Pack k-point meshes with densities of at least 3 Å.

### 3.3. Sound Propagation Studies

Direction-dependent phase velocities, group velocities, power-flow angles, and enhancement factors based on the stiffness tensor of a solid were calculated using Christoffel v1.2.1, a Python 2.7.16 tool for calculating sound velocities of a solid [60].

## 4. Conclusions

This study continues the investigation into the origin of the thermosalient effect in the anticholinergic agent oxitropium bromide, with a particular focus on thermal anisotropy, elastic properties, and sound propagation in this material leading to the thermosalient effect. Like other thermosalient materials, OXTB exhibits remarkably large anisotropic thermal expansion characterized by extremely high values of the linear thermal expansion coefficients. Notably, the low-temperature Form A of OXTB demonstrates NTE along the *c*-axis. This highlights the critical role of anisotropic thermal expansion, including NTE, in driving the thermosalient effect. By creating uneven internal stress within the crystal during heating, NTE amplifies anisotropy by intensifying dimensional mismatches across crystallographic axes. This pronounced thermal response destabilizes the crystal structure, triggering abrupt phase transitions that release mechanical energy, manifesting as characteristic leaps or movements.

In our previous study [23], we hypothesized that negative compressibility might be a key factor essential for the thermosalient effect. However, this work demonstrates that OXTB does not exhibit negative compressibility, challenging our earlier assumption. Instead, the results point to the high anisotropy of thermal expansion as the primary prerequisite for thermosalience.

In addition, the calculation of elastic constants for OXTB allowed us to determine key properties of sound propagation within the material, which, to the best of our knowledge, is the first study of sound propagation in thermosalient crystals. These findings for the first time reveal a preferred path for compressive wave propagation and energy transfer, indicating enhanced rigidness along this direction, which coincides with the direction of NTE. This facilitates the efficient release of accumulated strain energy, supporting the idea that the thermosalient phase transition results from the collective motion of molecules. It further confirms that the thermosalient phase transition is the direct consequence of the collective motion of the molecules, leading to the sudden release of the strain accumulated in the crystal lattice during thermal expansion.

The results of this study suggest that a material’s highly anisotropic thermal expansion can serve as a predictive indicator of its thermosalient behavior. This finding provides a valuable criterion for identifying new thermosalient materials more efficiently, potentially accelerating their discovery and facilitating the development of materials with tailored mechanical properties for practical applications.

## Figures and Tables

**Table 1:** Thermal expansion coefficients *α_i_* along principal axis *x_i_* (*i* = 1, 2 and 3) for OXTB low-temperature Form A determined from in situ variable-temperature X-ray diffraction.

		Component of *x_i_* Along the Crystallographic Axes		
Principal Axis, *i*	$\alpha_{i}\;(\times 10^{-6}K^{-1}$) for Form A	a	b	c
1	−47 (2)	0	0	1
2	55 (3)	1	0	0
3	117 (4)	0	1	0

**Table 2:** Thermal expansion coefficients *α_i_* along principal axis *x_i_* (*i* = 1, 2, and 3) for OXTB high-temperature Form B determined from in situ VTXRPD.

		Component of *x_i_* Along the Crystallographic Axes		
Principal Axis, *i*	$\alpha_{i}\;(\times 10^{-6}K^{-1}$) for Form A	a	b	c
1	21 (3)	0	1	0
2	18 (1)	0	0	1
3	104 (4)	1	0	0

**Table 3:** Principal compressibility *K_i_* for OXTB Form A along the principal axes *x_i_* (*i* = 1, 2 and 3) determined from DFT stiffness matrix calculations.

		Component of *x_i_* Along the Crystallographic Axes		
Principal Axis, *i*	*K_i_* (TPa^−1^) for Form A	a	b	c
1	15.8	1	0	0
2	26.2	0	1	0
3	12.9	0	0	1

**Table 4:** Anisotropy parameters for OXTB Form A calculated from elastic constants.

	Anisotropy Parameters		
	*A^L^*	*A^U^*	*A^C^*
Form A	0.20945	0.52453	0.045754

## Data Availability

Data are contained within the article.
